# PageRank versatility analysis of multilayer modality-based network for exploring the evolution of oil-water slug flow

**DOI:** 10.1038/s41598-017-05890-0

**Published:** 2017-07-14

**Authors:** Zhong-Ke Gao, Wei-Dong Dang, Shan Li, Yu-Xuan Yang, Hong-Tao Wang, Jing-Ran Sheng, Xiao-Fan Wang

**Affiliations:** 1 School of Electrical and Information Engineering, Tianjin University, Tianjin, 300072 China; 2 Department of Automation, Shanghai Jiao Tong University, and Key Laboratory of System Control and Information Processing, Ministry of Education of China, Shanghai, 200240 China

## Abstract

Numerous irregular flow structures exist in the complicated multiphase flow and result in lots of disparate spatial dynamical flow behaviors. The vertical oil-water slug flow continually attracts plenty of research interests on account of its significant importance. Based on the spatial transient flow information acquired through our designed double-layer distributed-sector conductance sensor, we construct multilayer modality-based network to encode the intricate spatial flow behavior. Particularly, we calculate the PageRank versatility and multilayer weighted clustering coefficient to quantitatively explore the inferred multilayer modality-based networks. Our analysis allows characterizing the complicated evolution of oil-water slug flow, from the opening formation of oil slugs, to the succedent inter-collision and coalescence among oil slugs, and then to the dispersed oil bubbles. These properties render our developed method particularly powerful for mining the essential flow features from the multilayer sensor measurements.

## Introduction

The exploration of oil-water flows, which cannot be modeled via a strict mathematical formula, has perplexed lots of researchers due to the complicated but significant flow behaviors arising from the various irregular spatial flow structures. Particularly, oil-water slug flow (D OS/W), one ubiquitous flow structure, has greatly exacerbated the complexity of oil-water flows. The diverse spatial-temporal configurations of oil slugs result in many special and characteristic dynamical flow behaviors, which exhibit the features of randomness, instability and instantaneity. The characterization of the abundant oil-water flow structures and behaviors underlying the evolution of oil-water slug flow represents a challenging and important research topic, which contributes to the understanding of the heat and mass transfer characteristics.

Different to the gas-liquid two-phase flow^1^, oil-water flows exhibit more complicated flow behaviors due to the existence of more complicated interfacial effect and relative motion among flow media. On account of the significant importance and increasingly challenging complexity, the study of oil-water flows has attracted a great deal of research interests and brought us many important achievements. In the past, based on the experimental measurements, many analysis techniques, such as time-frequency analysis^2^, power spectral density^3^, chaotic time series analysis^4, 5^, RQA and PCA analysis^6^ and support vector machine^7^, etc., have been successfully applied on multiphase flow. However, although these achievements have been made, the characterization of the spatial complicated flow behaviors underlying the evolution of oil slugs is still blurred and limited. In order to acquire the microcosmic flow information and reveal the spatial flow structures, we technically develop a double-layer distributed-sector conductance sensor (DLDSC Sensor). Moreover, neoteric multivariate data fusion theory is required to extract the effective information for characterizing the spatial complicated flow behaviors.

Complex network^8, 9^, serving as a powerful analytical framework for characterizing complex dynamics, has undergone a brilliant development in recent years. Plenty of achievements coming from diverse fields have demonstrated that complex network can efficiently cope with structural and dynamical problems of complex systems^10–20^. Quite recently, complex network analysis of time series^21–27^ has also gotten a lot of attention. Ref. 28 presents a review of complex network analysis of time series. Specifically, in view of that many real-world systems exhibit multiple characteristics among a same set of components^29–33^, large attentions have been focused on the multilayer network^34–36^, where a set of nodes simultaneously exist in multiple network layers with different types of edges. This burgeoning network framework has exhibited its strong capacity in exploring the structure, dynamics and function of diverse complex systems involving epidemic spreading^37^, brain system^38^, transportation system^39^ and social system^40^.

In this paper, aiming at characterizing the detailed flow structures and complicated spatial flow behaviors underlying the evolution of oil-water slug flow, we first carry out oil-water flow experiments and use our designed DLDSC Sensor system to acquire the abundant spatial-temporal flow information. And then we construct the multilayer modality-based network (MMBN) from the double-layer multi-channel measurements, where the modality determined in terms of the correlation between short-term measurements has close-knit connection with the flow structure. Moreover, we calculate the PageRank versatility^41^ and multilayer weighted clustering coefficient, respectively, for all derived MMBNs. PageRank versatility allows characterizing the multilayer network from the perspective of node versatility. The results validate the compact connection between the local features of MMBNs and the intricate spatial flow behaviors in the oil-water slug flow. These properties render our developed method particularly useful for probing the intrinsic flow behaviors from multilayer sensor measurements.

## Experiments

**Figure 1:**
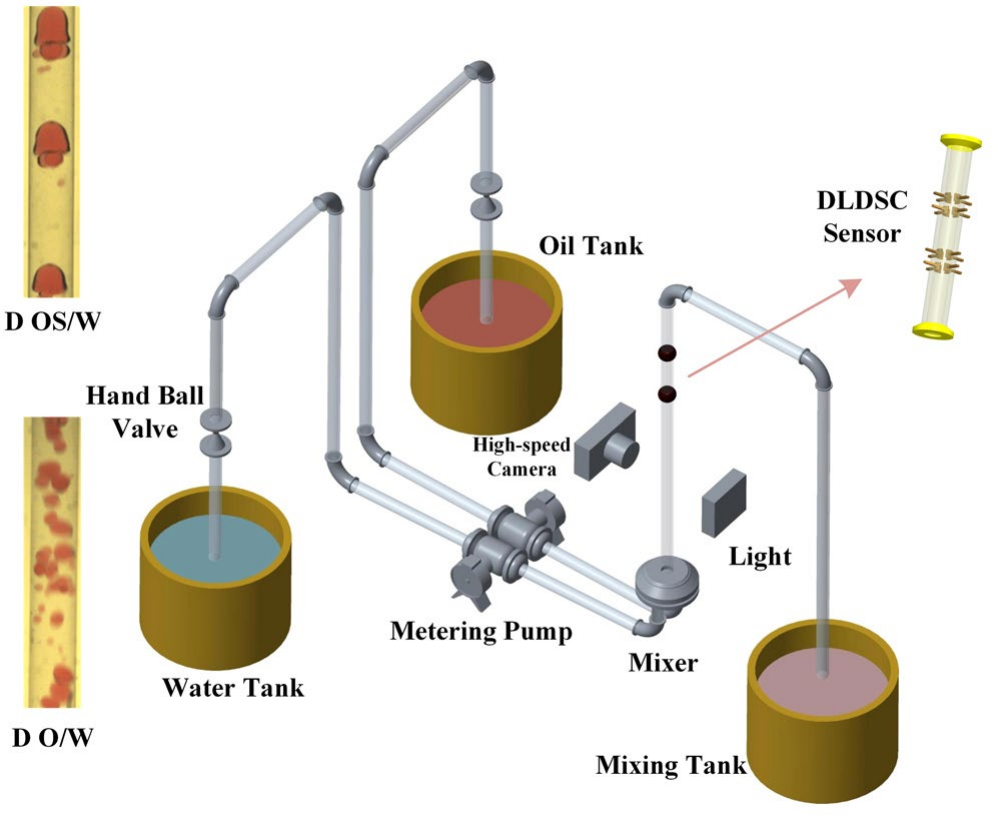
Schematic of vertical upward oil-water two-phase flow loop facility.

In order to capture the spatial flow information of the oil-water slug flow, we design a DLDSC Sensor and conduct oil-water flow experiments in a vertical 20-mm-diameter pipe. As displayed in Fig. 1, the flow loop facility mainly contains three parts: namely, three tanks for storing tap-water, oil and mixture fluid, respectively; the pipe and valves for connecting and building the flow loop; the designed sensor (denoted as DLDSC Sensor) and a high-speed video camera for capturing the requisite spatial flow image information. The DLDSC Sensor, consisting of the up-structure sectors and down-structure sectors, is specially designed for acquiring spatial oil-water flow information from different pipe positions. In our experiments, the water-cut and total flow velocity are set as two pivotal parameters to adjust and generate flow conditions. Note that, we increasingly study 8 discrete total flow velocities in the range of 0.0184 m/s–0.2579 m/s for each specified water-cut and the water-cut is set as 60%, 70%, 80%, 82% and 84%, respectively. For each flow condition, we first pump the tap-water and oil into the vertical pipe via two peristaltic metering pumps, respectively. Before the mixture flow arrives at the measuring section, namely the sensor position, oil and water adequately mix with each other and reach a stable flow state (flow structure). Then the double-layer multi-channel measurements of oil-water flows are acquired via the DLDSC Sensor system. Meanwhile, the snapshots from high-speed video camera are used for helping classify different experimental flow structures. Finally, the mixture flow is drained into the mixing tank where oil and water would naturally separate due to the action of gravity.

### Exploring the evolution of oil-water slug flow via MMBN

**Figure 2:**
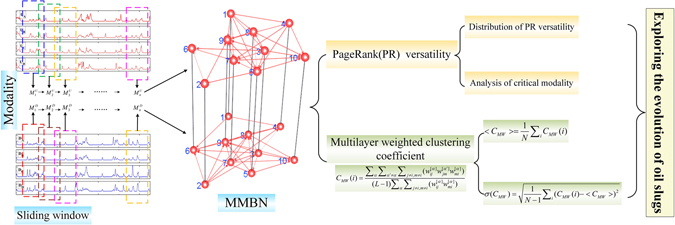
A schematic diagram of our analytical framework for exploring the evolution of oil slugs. We give a simplified MMBN with 10 nodes here for presentation.

Based on the experimental double-layer multi-channel measurements, we infer MMBN for each experimental flow condition, aiming at uncovering the intricate spatial flow structures and dynamical flow behaviors associated with the evolution of oil slugs in oil-water flows. A schematic diagram of our method is presented in Fig. 2. We emphasize here that each modality in our MMBNs exactly corresponds to one specific configuration of oil and water phase in the experimental circular pipe.

From the perspective of node versatility, PageRank versatility^41^ successfully serves as a good descriptor of dynamical aspects of complex system. We here use a 4th-order tensor^42^ ${M}_{j\beta }^{i\alpha }$ to encode a directed, weighted connection between node *i* from layer *α* to any other node *j* in layer *β* (including *β* = *α*). The network has *L* layers and each layer has *N* nodes. We indicate with ${D}_{j\beta }^{i\alpha }$ the strength tensor whose entries are all zeros, except for *i* = *j* and *α* = *β* where the entries are given by the out-degree ${s}_{i\alpha }$(including intra-and inter-layer links) of node *i* in layer *α*. And ${\tilde{D}}_{j\beta }^{i\alpha }$ is the tensor whose entries are the inverse of the non-zero entries of ${D}_{j\beta }^{i\alpha }$. For the multilayer network ${M}_{j\beta }^{i\alpha }$, the transition probabilities of jumping between pairs of nodes and switching between pairs of layers can be denoted as ${T}_{j\beta }^{i\alpha }$:

1 $${T}_{j\beta }^{i\alpha }={M}_{j\beta }^{k\gamma }{\tilde{D}}_{k\gamma }^{i\alpha },$$

which allows to provide a one-to-one relationship between the intra-and inter-layer links in ${M}_{j\beta }^{i\alpha }$ with the transition probabilities in ${T}_{j\beta }^{i\alpha }$. We use ${{\bf{A}}}_{j\beta }^{i\alpha }$ to denote the appearance of dangling nodes which have no forward links. Particularly, if node *i* in layer *α* is a dangling node, we set ${{\bf{A}}}_{j\beta }^{i\alpha }$ to 1 for all variational *j* and *β*, otherwise 0. Note that a dangling node corresponds to a row in ${M}_{j\beta }^{i\alpha }$ with all entries equal to 0, while a row in ${{\bf{A}}}_{j\beta }^{i\alpha }$ with all entries equal to 1. To eliminate the interference of dangling node, we conduct the following operation:

2 $${S}_{j\beta }^{i\alpha }={T}_{j\beta }^{i\alpha }+\frac{{{\bf{A}}}_{j\beta }^{i\alpha }}{NL}.$$

This means that the random surfer escapes from the dangling page by jumping to a randomly chosen page. ${S}_{j\beta }^{i\alpha }$ is called resulting matrix.

Assuming that the walker jumps to a neighbor with rate *r* and teleports to any other nodes in the multilayer network ${M}_{j\beta }^{i\alpha }$ with rate 1−*r*, we construct the transition tensor ${R}_{j\beta }^{i\alpha }$ (a rank-4 tensor):

3 $${R}_{j\beta }^{i\alpha }=r{S}_{j\beta }^{i\alpha }+\frac{(1-r)}{NL}{u}_{j\beta }^{i\alpha },$$

where ${u}_{j\beta }^{i\alpha }$ is a rank-4 tensor with all components equal to 1. We use *r* = 0.85 as in the classical PageRank algorithm. Specifically, mathematically, the iterative procedure can be replaced via calculating the largest eigenvalue and corresponding eigenvector of the adjacency matrix. So then we calculate the eigentensor Ω_*iα*_ (related to the largest eigenvalue) of the transition tensor ${R}_{j\beta }^{i\alpha }$, denoting the steady-state probability to find the walker at node *i* of layer *α*. The multilayer PageRank versatility is obtained by contracting the layer index of the eigentensor with the 1−vector *u* ^*α*^: *PR*_*i*_ = Ω_*iα*_ *u* ^*α*^, i.e., summing up over layers.

**Figure 3:**
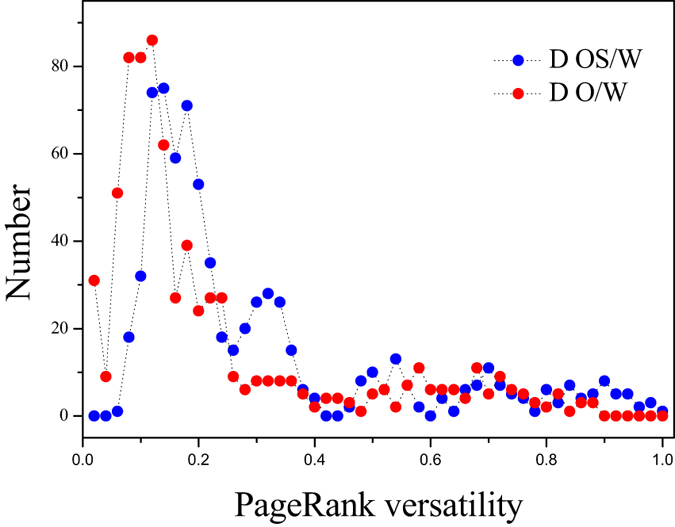
The PageRank versatility distribution in the derived MMBN.

**Figure 4:**
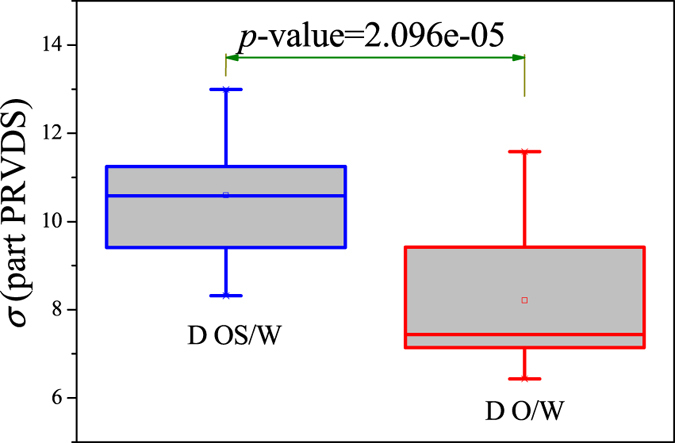
Boxplots of the standard deviation of the part PageRank versatility distribution series (part PRVDS) for oil-water slug flow and bubble flow.

The PageRank versatility distribution for two different flow conditions, belonging to two typical oil-water flow patterns respectively, are displayed in Fig. 3. The interval of the PageRank versatility is 0.02. The distribution of these two typical flow patterns display similar variation trend, arising from the fact that they all belong to oil-water flows with water as the continuum. For instance, they all present an obvious peak at the start of the distribution. This phenomenon can be considered as the mirror of the common and mutual flow structures in both flow patterns, such as continuous water with tiny oil droplets. However, we also find that different significant fluctuations appear after the peak, attributed to the fact that each flow pattern has its typical and special flow structures and behaviors. In order to further explore these significant fluctuations in the complicated evolution of oil slugs, we calculate the standard deviation of the part PageRank versatility distribution series (i.e., the PageRank versatility distribution series except for the first 9 points in that these 9 points in the peak correspond to the common features of oil-in-water flows) for each flow condition. And the results for these two flow patterns are shown in Fig. 4 via boxplots.

Clustering coefficient, serving as one of the most popular network measures, can effectively quantify the tendency of nodes to form triangles. According to ref. 43, multilayer weighted clustering coefficient (*C*_*MW*_) can be defined and then applied to our derived MMBNs. For a multiplex network M = {*M*_1_, *M*_2_, …, *M*_*L*_}, where the adjacency matrix of each layer *M*_*k*_ is denoted as *W*_*k*_ = $({w}_{ij}^{[k]})\in {{\bf{R}}}^{N\times N}$, the *C*_*MW*_ of node *i* is defined as:

4 $${C}_{MW}(i)=\frac{{\sum }_{\alpha }{\sum }_{{\alpha }^{^{\prime} }\ne \alpha }{\sum }_{j\ne i,m\ne i}({w}_{ij}^{[\alpha ]}{w}_{jm}^{[{\alpha }^{^{\prime} }]}{w}_{mi}^{[\alpha ]})}{(L-1){\sum }_{\alpha }{\sum }_{j\ne i,m\ne i}({w}_{ij}^{[\alpha ]}{w}_{mi}^{[\alpha ]})}(j\ne m).$$

We note here that *L* = 2 in our MMBN. The concept of two-triangle is defined as follows: a triangle which is formed by an edge belonging to one layer and two edges belonging to a second layer^43^. Averaging this quantity over all the nodes in the network, we get the multilayer average weighted clustering coefficient:

5 $$ < {C}_{MW} > =\frac{1}{N}{\sum }_{i}{C}_{MW}(i).$$

Correspondingly, standard deviation of *C*_*MW*_(*i*) can be presented as:

6 $$\sigma ({C}_{MW})=\sqrt{\frac{1}{N-1}{\sum }_{i}{({C}_{MW}(i)- < {C}_{MW} > )}^{2}},$$

where the number of nodes in each layer *M*_*k*_ is *N*. We note that <*C*_*MW*_> provides an entire evaluation for the connectivity of the considered MMBN, and the *σ*(*C*_*MW*_) can exactly reflect the discrepancy among the modalities (i.e., nodes).

**Figure 5:**
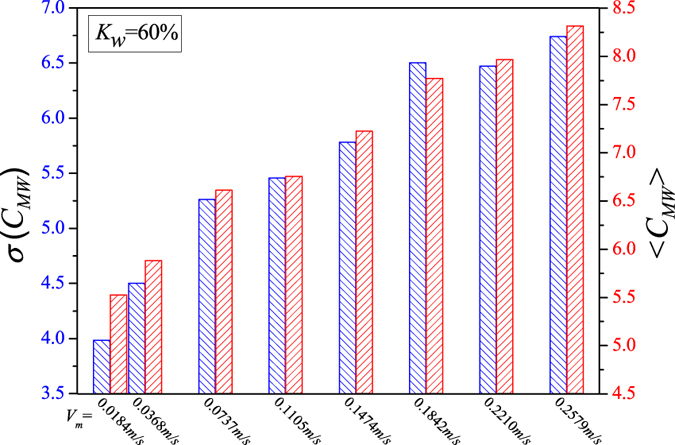
<*C*_*MW*_> and *σ*(*C*_*MW*_) for flow conditions with different total flow velocities (*V*_*m*_) when water-cut *K*_*w*_ = 60%.

**Figure 6:**
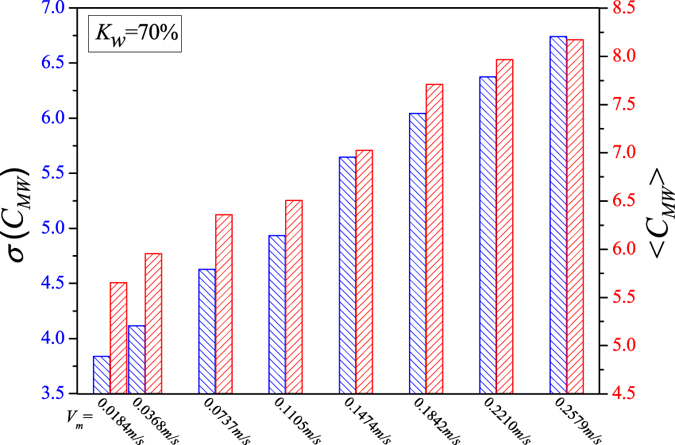
<*C*_*MW*_> and *σ*(*C*_*MW*_) for flow conditions with different total flow velocities (*V*_*m*_) when water-cut *K*_*w*_ = 70%.

**Figure 7:**
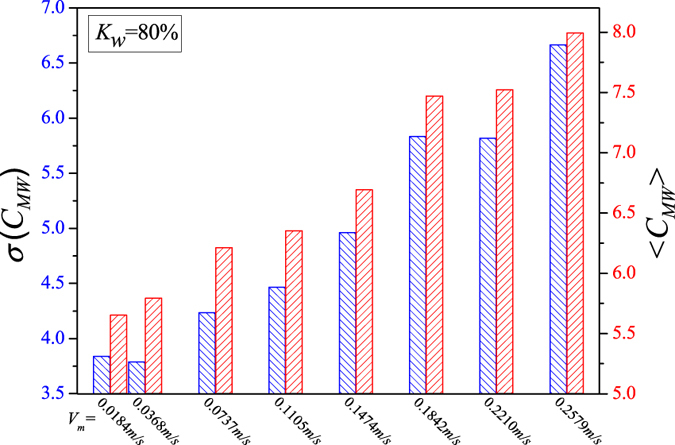
<*C*_*MW*_> and *σ*(*C*_*MW*_) for flow conditions with different total flow velocities (*V*_*m*_) when water-cut *K*_*w*_ = 80%.

**Figure 8:**
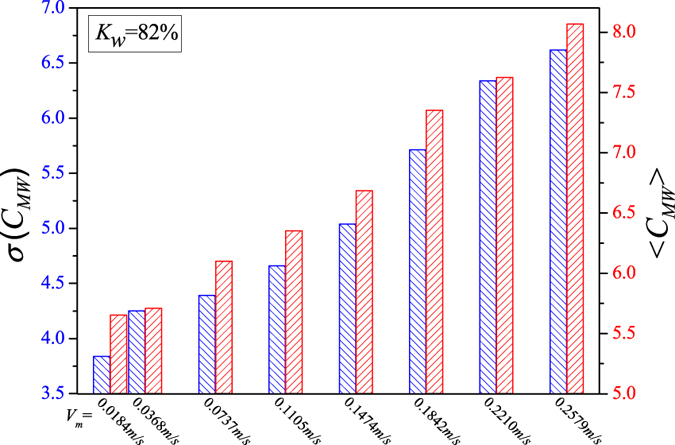
<*C*_*MW*_> and *σ*(*C*_*MW*_) for flow conditions with different total flow velocities (*V*_*m*_) when water-cut *K*_*w*_ = 82%.

**Figure 9:**
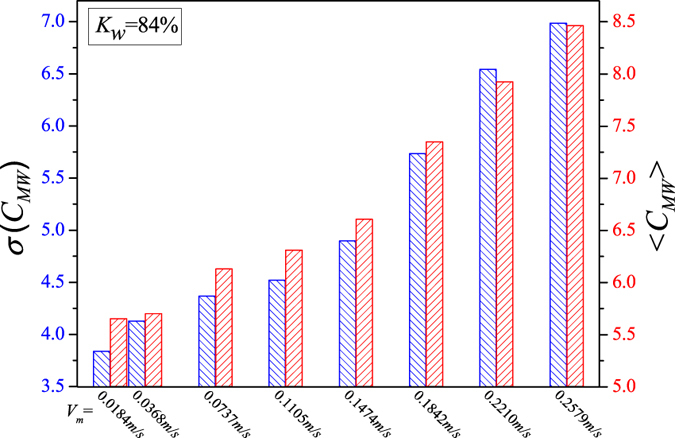
<*C*_*MW*_> and *σ*(*C*_*MW*_) for flow conditions with different total flow velocities (*V*_*m*_) when water-cut *K*_*w*_ = 84%.

We display the calculated network measures in Figs 5, 6, 7, 8 and 9 for different fixed water-cuts, respectively. The distributions of network measures provide a quantitative mapping of the evolution of oil slugs in oil-water flows. In concrete terms, when the total flow velocity is low, the mixture flow appears as oil-water slug flow. As can be seen in Figs 5, 6, 7, 8 and 9, <*C*_*MW*_> and *σ*(*C*_*MW*_) both present small values. The small <*C*_*MW*_> means that modalities are hard to repeat in adjacent steps and two-triangles are difficult to form, which implies that spatial flow structures in the considered flow conditions are desultory, namely, the mixture flow presents strong transient and the flow structures experience a large change even during a short period. As a result, different and diverse flow structures appear in such a flow pattern, range from cap-shaped oil slugs to very fine oil droplets. And the modalities, reflecting the oil-water spatial transient flow structures, have almost the same probability to appear in such a situation, which can be validated via the small value of *σ*(*C*_*MW*_). All these are consistent with the intermittent quasi-periodic flow characteristic and heterogeneity of flow structures in the oil-water slug flow. In particular, the large value of standard deviation of the part PageRank versatility distribution series for slug flow shown in Fig. 4 also quantitatively indicates the heterogeneity in such a flow pattern. With the continuous increase of total flow velocity (*V*_*m*_), <*C*_*MW*_> and *σ*(*C*_*MW*_) both become larger, as shown in Figs 5, 6, 7, 8 and 9. These changes demonstrate that the flow behavior becomes more random and the heterogeneity of flow structures becomes weakened. These result from the fact that the small oil slugs and oil droplets become more difficult to polymerize into large oil slugs with the increase of *V*_*m*_. When the total flow velocity reaches to a higher value, <*C*_*MW*_> and *σ*(*C*_*MW*_) continually become larger. In particular, the standard deviation of the part PageRank versatility distribution series for oil-water bubble flow (D O/W) present relatively small values, which demonstrate that the spatial heterogeneous distribution of oil slugs is gradually weakened and the spatial local flow behaviors start exhibiting stochastic features. Based on the snapshots acquired from high-speed camera, the flow structures gradually become dispersed oil bubbles flowing in a water continuum. Namely, the oil slugs gradually break into stochastic oil droplets. In addition, based on the standard deviation of the part PageRank versatility distribution series for different flow patterns, we calculate the *p*-value based on the t-test, as shown in Fig. 4. The *p*-value is obviously smaller than 0.05 (a benchmark for determining the significant difference from the *p*-value), indicating that these two oil-water flow patterns have obvious difference from the perspective of PageRank versatility.

**Figure 10:**
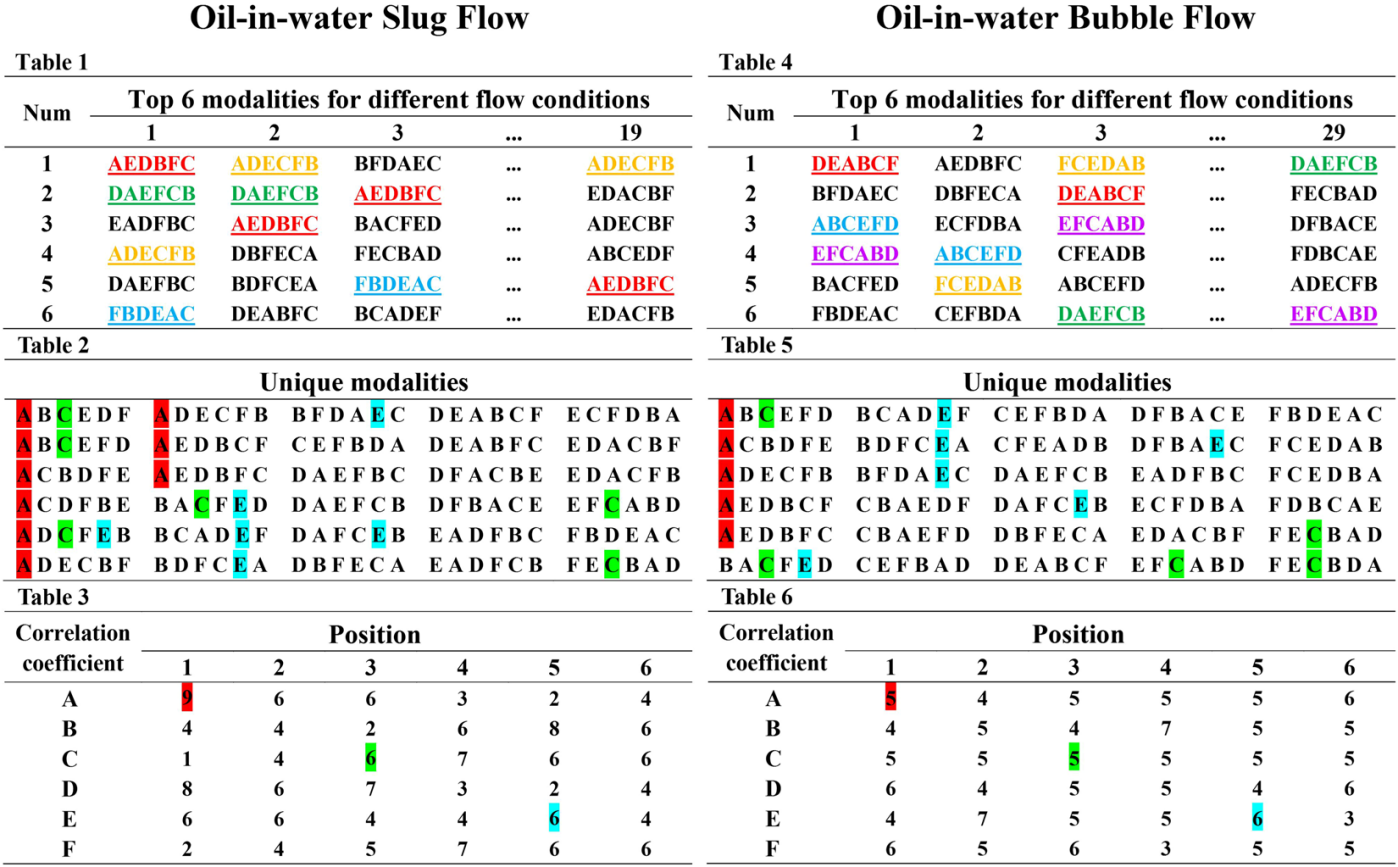
The analysis of the critical modalities in oil-water slug flow and bubble flow. Table 1 presents the top 6 critical modalities in 19 oil-water slug flow conditions; Table 2 shows the unique modalities from Table 1 (i.e., all the modalities that appear in Table1 are listed only once in Table 2); and we break down the unique modalities in Table 2 and map the results into Table 3, where the element *A1* in the *A* row and the *1st* column shows the statistic of the correlation coefficient *A* in the *1st* position in unique modalities. And similarly, Table 4, Table 5, and Table 6 are for 29 oil-water bubble flow conditions.

From the perspective of clustering coefficient and PageRank versatility, we effectively uncover and present the complicated evolution of oil slugs, from the opening formation of oil slugs, to the succedent inter-collision and coalescence among oil slugs, and then to dispersed oil bubbles. In short, for a specified water-cut (*K*_*w*_), the increase of total flow velocity (*V*_*m*_) from 0.0184 m/s to 0.2579 m/s leads to a series of complicated changes of flow states underlying the evolution of oil-water slug flow. One step closer, we study the top 6 critical modalities, determined in terms of the PageRank versatility, in each flow condition. The statistical and analytical processes refer to Fig. 10. The majority critical modalities are shared in both flow patterns, as shown in Table 2 and Table 4 of Fig. 10, which agrees with our above-mentioned analysis that there exist many common flow structures between these two flow patterns arising from continuous water phase. However, some special critical modalities only appear in specific flow pattern declaring the differences between the oil-water slug flow and bubble flow. Then we break down the critical modalities and figure out some interesting phenomena. We note that a modality is a permutation of 6 different correlation coefficients (see methodology part for details). Particularly, in the slug flow, there are significant differences (e.g., *A1* is 9, while *D5* is 2) in the occurrence frequency of different correlation coefficients at different locations, while in the bubble flow, the number of each correlation coefficient in each position is relatively uniform and all close to 5. All these features again validate the heterogeneous in the slug flow while the randomness in the bubble flow.

## Discussion

How a big oil slug can break into oil droplets with diverse sizes and shapes constitutes a fundamental but challenging problem of great importance in field of oil exploration. Based on the double-layer multivariate measurements acquired from our designed DLDSC Sensor system, we infer the MMBN and correspondingly calculate the PageRank versatility and multilayer weighted clustering coefficient, aiming at quantitatively characterizing the detailed flow structures and complicated spatial flow behaviors underlying the evolution from oil-water slug flow to bubble flow. The results demonstrate that these two multilayer network measures can effectively characterize the complicated evolution of oil-water slug flow. Our method provides a novel way for fusing the spatial multivariate sensor measurements, which allows characterizing the complicated dynamical behaviors of complex systems.

## Methods

### Multilayer modality-based network

As a development of previously modality transition-based network theory^1^, we infer the multilayer modality-based network (MMBN) from the double-layer multivariate measurements. Spatial complicated transitional behaviors of oil-water flows are efficiently mapped into the intra- and inter-layer edges in MMBN.

In concrete terms, we firstly partition the double-layer measurements via a sliding window (length 20), which slides along time by a step of 1. As shown in Fig. 2, the resulting sub-time series in each window consist of 4 channels for each layer, respectively. And we then determine the modality from the sub-time series in each window as follows:

Firstly, we calculate the correlation between any two sub-time series by the following equation

7 $${r}_{xy}=\frac{{\sum }_{i=1}^{{n}^{^{\prime} }}({x}_{i}-\overline{x})({y}_{i}-\overline{y})}{\sqrt{{\sum }_{i=1}^{{n}^{^{\prime} }}{({x}_{i}-\overline{x})}^{2}}\sqrt{{\sum }_{i=1}^{{n}^{^{\prime} }}{({y}_{i}-\overline{y})}^{2}}},$$

where *n*′ = 20 and all six correlation coefficients including *r*_12_,*r*_13_,*r*_14_,*r*_23_,*r*_24_ and *r*_34_ are set as the elements of a modality:

$${r}_{12}\to A,{r}_{13}\to B,{r}_{14}\to C,{r}_{23}\to D,{r}_{24}\to E,{r}_{34}\to F.$$

We then rank the six correlation coefficients in one layer incrementally to obtain a modality. For instance, if *A* < *C* < *B* < *D* < *F* < *E*, we obtain the modality *ACBDFE*. The number of all possible modalities is equal to the strings of the permutations of *A*,*B*,*C*,*D*,*E* and *F*, i.e., 6! = 720. So we get two modality sequences, ${M}_{i}^{U},\,i=1,2,\ldots ,n$ and ${M}_{i}^{D},\,i=1,2,\ldots ,n$ (as shown in Fig. 2), corresponding to the double-layer measurements, respectively. Based on these two modality sequences, the directed and weighted intra- and inter-layer edges in MMBN are determined in terms of the direction and times of the transition among modalities. We define each modality as a node and set all the nodes in a fixed and identical order in all layers. Taking the up-layer *W* ^*U*^ of the MMBN as an example, we construct a directed intra-edge from ${M}_{i}^{U}$ (e.g., modality *α*) to ${M}_{i+1}^{U}$ (e.g., modality *β*) in *i-*th step and the ${W}_{\alpha \beta }^{U}$ pluses one. So repeated transition between two different modalities (e.g., *α* and *β*) in different steps would let ${W}_{\alpha \beta }^{U}$ have a large value. Self-transitions are excluded. And if ${M}_{i}^{U}={M}_{i}^{D},\,{M}_{i+1}^{U}={M}_{i+1}^{D}$, we construct a bidirectional inter-edge between ${M}_{i}^{U}$ and ${M}_{i}^{D}$. The above procedure allows us to infer a two-layer multilayer network with 720 nodes in each layer. In order to reduce the effect of unavoidable noise arising from the non-characteristic flow in different flow patterns, we first remove the edges with weight 1 and then delete the isolated nodes in the resulting MMBNs. This operation allows maintaining and highlighting the critical modalities for further exploration of spatial flow structures and behaviors.
